# Megavoltage intrafraction monitoring and position uncertainty in gimbaled markerless dynamic tumor tracking treatment of lung tumors

**DOI:** 10.1002/mp.17740

**Published:** 2025-04-03

**Authors:** Marco Serpa, Tobias Brandt, Simon K. B. Spohn, Andreas Rimner, Christoph Bert

**Affiliations:** ^1^ Department of Radiation Oncology Universitätsklinikum Erlangen, Friedrich‐Alexander‐Universität Erlangen‐Nürnberg Erlangen Germany; ^2^ Department of Radiation Oncology, Medical Center ‐ University of Freiburg, Faculty of Medicine University of Freiburg, German Cancer Consortium (DKTK) partner site DKTK‐Freiburg Freiburg Germany

**Keywords:** intrafraction monitoring, markerless dynamic tumor tracking, megavoltage tracking

## Abstract

**Background:**

The clinical realization of markerless dynamic tumor tracking (MLDTT) has prompted a new paradigm shift to intrafraction imaging‐based quality assurance (QA). During MLDTT treatment using a gimbaled accelerator, the megavoltage (MV) imager serves as an independent system to leverage real‐time intrafraction monitoring. Soft‐tissue feature tracking has shown promise for tumor localization in confined MV projections, but studies demonstrating its application in clinical MLDTT treatments are scarse.

**Purpose:**

To validate MV image‐based dense soft‐tissue feature tracking for intrafraction position monitoring of lung tumors during MLDTT stereotactic body radiotherapy (SBRT), and report on the resolved geometric uncertainty.

**Methods:**

Ten non‐small cell lung cancer (NSCLC) patients underwent MLDTT‐SBRT using a commercial gimbal‐based system. During treatment, beam's‐eye‐view (BEV) projection images were captured at ∼3 frames s^−1^ (fps) using the electronic portal imaging device (EPID). MV sequences were streamed to a research workstation and processed off‐line using a purpose‐built algorithm, the soft‐tissue feature tracker (SoFT). Both the tumor and dynamic field aperture position were automatically extracted in the pan and tilt directions of the gimbaled x‐ray head, corresponding to the in‐plane lateral and longitudinal direction of the imager, and compared to ground truth manual tracking. The success, percentage of fields producing an output, and performance of MV tracking under the presence/absence of anatomy‐related obstruction and multi‐leaf collimator (MLC) occlusion were quantified, including three‐dimensional conformal (3DCRT) and step‐and‐shoot intensity modulated radiotherapy (IMRT) deliveries. In addition, the geometric uncertainty of MLDTT treatment was estimated as the difference between field aperture and target center position in the BEV. The standard deviation of systematic (*Σ*) and random (*σ*) errors were determined.

**Results:**

MV tracking was successful for 89.7% of (unmodulated) 3DCRT fields, as well as 82.4% of (modulated) control points (CPs) and subfields (SFs) for IMRT and field‐in‐field 3DCRT deliveries. The MV tracking accuracy was dependent on the traversed anatomy, tumor visibility, and occlusion by the MLC. The mean MV tracking accuracy was 1.2 mm (pan) and 1.4 mm (tilt), and a resultant 2D accuracy of 1.8 mm. The MV tracking performance within 2 mm was observed in 92.1% (pan) and 86.6% (tilt), respectively. The mean aperture‐target positional uncertainty smaller than 3 mm/5 mm was observed in 94.4/97.9% (pan) and 89.6/96.7% (tilt) of the time. The group *Σ* and *σ* were 0.5 mm/0.8 mm (pan), and 0.7 mm/1.2 mm (tilt), compared to 0.3 mm/0.5 mm (pan), and 0.6 mm/0.9 mm (tilt) based on the manual ground truth.

**Conclusion:**

MV imaging coupled with the soft‐tissue feature tracker algorithm constitutes a valuable non‐invasive method for independent intrafraction surveillance. Tracking multiple features has shown the potential to improve position estimation, notwithstanding obstruction, and occlusion challenges, facilitating the quantification of the geometric uncertainty of MLDTT treatment.

## 1 INTRODUCTION

Intrafraction organ motion, particularly respiratory motion, constitutes a major limiting factor to the precise application of highly conformal doses to pulmonary lesions in external beam radiotherapy.^1^ Lung tumor motion is complex, patient‐/fraction‐specific, and unpredictable.^2^ Common practice is the motion‐inclusive internal target volume (ITV)‐based approach.^3^ This inevitably results in enlarged treatment apertures and additional doses to surrounding healthy tissue. Even then, geometric misses may still occur. Motion at treatment may be significantly different and exceed that measured during a single pre‐treatment four‐dimensional computed tomography (4DCT) simulation^4^, ^5^, ^6^ and cone beam (4DCBCT).^7^ Recently, the AAPM TG324 acknowledged the continuing importance and requirement of motion management strategies.^8^ Dynamic tumor tracking (DTT) is one such technique where real‐time adaptation is accomplished by repositioning the therapeutic beam continuously in response to tumor motion. Robotic,^9^ gimbaled,^10^, ^11^ and multi‐leaf collimator (MLC) tracking^12^ are clinically established^13^ with the access to magnetic resonance‐guidance technology on the rise.^14^ DTT may enable the ultimate means toward treatment volume reduction and dose escalation to ablative levels.^12^, ^13^ Most solutions rely primarily on implanted fiducials tracked in x‐ray fluoroscopy^9^, ^10^, ^11^ or electromagnetic transponders^12^ for target localization. While there is a general consensus supporting implanted fiducials usage such for liver^15^ or prostate^16^ treatments, this is not widely accepted for pulmonary lesions. Marker implantation possess risks (e.g., pneumothorax).^17^ Moreover, x‐ray imaging has the inherent disadvantage of exposing the patient to additional dose levels.^18^

To date, markerless dynamic tumor tracking (MLDTT) has become available on three commercial photon external beam radiotherapy platforms: the CyberKnife, *XSight Lung* ^19^ (Accuray, Sunnyvale, CA, USA), the RadiXact Synchrony^20^ (Accuray, Sunnyvale, USA), and the Vero4DRT (Mitsubishi Heavy Industries (MHI), and BrainLab, Munich, Germany).^2^ ^1^ All three systems are characterized as hybrid‐based in that external (optical) tracking and some sort of correlation model (CM)—established correlation between internal (tumor) and external (breathing) motion, usually calculated prior to treatment—are combined to reposition the irradiation beam in real‐time. A key aspect of hybrid‐based DTT technologies^9^, ^10^, ^11^ is the stability of internal/external correlation.^22^, ^23^ The performance of MLDTT has been shown to be comparable to the conventional marker‐based DTT.^21^ However, it introduces additional failure modes into the delivery workflow, namely, target localization failure at treatment due to reduced tumor visibility and overlaying structures in orthogonal x‐ray images at certain gantry‐ring angle combinations.^21^ Moreover, there is no verification system independent of the accelerator hardware/tracking software, and errors in feedback loops (e.g., look‐ahead capabilities of CM, response), generic to most DTT technologies,^13^ are latent. Moreover, with hypofractionated regimes carried out in as few as three to five fractions, and the increasing evidence favoring single‐fraction schedules,^14^ undetected errors would have a large dosimetric impact. Therefore, the positional accuracy should be verified.^12^, ^13^, ^22^, ^23^

Intra‐treatment surveillance through direct soft‐tissue tumor localization using an independent system may ensure safety and increase confidence. Tumor position verification via megavoltage (MV) imaging is highly appealing, namely it does not expose the patient to additional dose,^24^ provides the dosimetrically most relevant perspective^25^ in the beam's‐eye‐view (BEV) with minimal extra burden to the workflow,^24^ and independent of the primary respiratory monitoring system. A number of marker‐free approaches have been proposed for lung tumor tracking in BEV projections, including single‐,^26^, ^27^ multi‐template^28^ registration, and feature tracking.^29^ Although some of these methods have been subjected to extensive performance analysis on large datasets, few have addressed issues of target obstruction and/or occlusion. Richter et al.^27^ and Yip et al.^30^ demonstrated their methods on MV projections acquired during ITV‐based three‐dimensional conformal radiotherapy (3DCRT). Obstructions and occlusions were either discarded^27^ or unaddressed.^30^ Obstruction may be caused by nearby organs (e.g., spine, ribs, diaphragm) or external structures (e.g., table‐top edges) along the propagation path of the therapeutic beam. MLC occlusion, on the other hand, restricts target localization within the confines of the BEV. Furthermore, in SBRT planning the emphasis is on using highly conformal fields to set high‐dose heterogeneity, centered in the target volume, while reducing the high‐dose volume to the edges. Additionally, with intensity‐modulated radiotherapy (IMRT) and volumetric‐modulated arc radiotherapy (VMAT) techniques routinely being used in cases where tumors are in the proximity of critical structures and fulfill dose constraints, target occlusions incurred by the MLC become unavoidable.

It has been suggested that multi‐template^28^ or ‐feature^29^ tracking may be advantageous over single‐template approach in that it can accommodate affine changes or non‐rigid deformations and potentially occlusions. We have previously proposed a MV image‐based soft‐tissue feature tracking method to address occlusions challenges.^31^ In terms of validation, the method was demonstrated under limited scenarios using phantom and few clinical datasets (nine sequences). In this study, we built on our previous work by including a larger, more diverse dataset comprising tumors of varying sizes, motion amplitudes, and locations. This study improves our previously presented method by: (i) performing an in‐depth analysis of the impact of anatomy‐ and/or MLC‐related obstruction/occlusion on MV tracking performance, (ii) presenting a potential approach for independent surveillance with complementary functionality enabling the detection of residual errors, and (iii) estimating the geometric uncertainty of clinical MLDTT SBRT.

## 2 METHODS AND MATERIALS

### 2.1 Patient cohort

Ten NSCLC patients treated with MLDTT using the Vero4DRT system between August 2018 and July 2022 were included. Note that, MLDTT is the prioritized treatment option for suitable lesions, namely well‐localized tumors exhibiting sufficient contrast in kilovoltage (kV) x‐ray fluoroscopy.^21^ For each case, three CT scans were acquired: one free breathing, one end‐exhale (EE), and one end‐inhale (EI) scan, using a Siemens Somatom Go. Open Pro. CT scanner (Siemens Healthcare AG, Erlangen, Germany). The PTV was defined as the union of the gross tumor volume (GTV) at EE phase and mid‐ventilation expanded by 5 mm.^23^ The EE CT was used for treatment planning using RayStation (RaySearch Laboratories AB, Stockholm, Sweden). Five to eight‐field 3DCRT and step‐and‐shoot IMRT plans were calculated comprising 73 gantry‐ring angle combinations coplanar and noncoplanar. Of these, 55/73 (75.3%) comprised 3DCRT and 18/73 (24.7%) IMRT fields (see Table 1). In addition, a backup plan was created for breath‐hold delivery at EE in the event MLDTT treatment failed. The fractionation scheme ranged from 5 to 7 Gy/fraction in 7–12 fractions, prescribed to the 80% isodose level.

**TABLE 1:** Summary of the patients, GTV characteristics,^a^ fields (per fraction) with (w) and without (wo) identified anatomy‐ and MLC‐related target obstruction/occlusion.

		GTV						Fields^b^															Number of		
Case	Plan	Location			Vol.	Amp.	Diam.							Anatomy Obs.^c^		MLC Occl.^d^			Occl. Level^f^				Fractions	Sequences	Frames
No.	Type	LR	U|M|L	AP	(cm^3^)	(mm)	(mm)	Main	[	SF	|	CP	]	wo	w	wo	w	^e^	I	II	III	IV	Used	Acquired	analyzed
30	3DCRT	R	L	A	1.5	12.2	1.2	5		–	|	–		3	2	5	–			–			5	25	1929
32	IMRT	R	L	P	71.5	13.6	6.8	6		–	|	13		2	4	–	6	5	2	2	3	1	7	42	1425
32b	IMRT							6		–	|	49		2	4	–	6	13	8	9	6	13	6	36	3845
34	3DCRT	L	U	A	22.9	4.6	3.7	6		3	|	–		1	5	3	3	0	1	0	0	2	8	64	2504
42	3DCRT	L	U	A	2.1	3.1	1.6	8		–	|	–		3	5	8	–			–			9	72	3257
43	3DCRT	L	L	P	3.7	45	1.8	7		2	|	–		4	4	6	1	0	0	0	1	0	1	8	396
44	IMRT	R	L	P	24.8	13.9	3.8	6		–	|	22		1	5	–	6	5	5	2	4	6	7	42	4086
45	3DCRT	R	U	A	3.1	3.4	1.7	7		1	|	–		1	7	7	1	0	0	0	1	0	10	80	3749
51	3DCRT	L	U	A	2.9	9.2	1.8	8		1	|	–		2	6	7	1	0	0	0	0	1	9	72	3755
64	3DCRT	R	U	A	2.4	7.8	1.6	7		–	|	–		1	6	7	–			–			11	77	4684
70	3DCRT	L	L	P	7.6	19.3	2.3	7		1	|	–		3	4	6	1	0	0	1	0	0	12	84	4440
Total								73	[	7	|	84	]	21	52	48	25	23	16	14	15	23	85	602	34 070

^a^ GTV location in the left‐right (LR) lung, upper‐middle‐lower (U|M|L) lobe, and anterior‐posterior (AP) position, peak–peak motion amplitude (Amp.), and diameter (diam.) in superior–inferior direction.

^b^ Total number of main, sub‐fields (SFs) for 3DCRT, and control points (CPs) for IMRT deliveries per fraction.

^c^ Number of (main) fields with anatomy‐related obstruction.

^d^ Number of (main) fields with MLC‐related occlusion.

^e^ Represents the number of un‐occluded SFs/CPs.

^f^ Represents the number of occluded SFs/CPs with (estimated) occlusion levels: I (slight), II (moderate), III (high), and IV (total).

### 2.2 MLDTT treatment

The Vero4DRT system features a compact 6‐MV linac on a two‐axis gimbal and orthogonal kV x‐ray imaging subsystem mounted in an O‐ring gantry.^10^ In the MLDTT workflow, marker implantation is no longer necessary to perform DTT. A detailed description of the MLDTT workflow can be found in previously published work.^21^ In brief, the patient is first positioned under laser guidance and skin markers. An infra‐red (IR) marker pad is placed on the abdomen to monitor the external breathing motion. This is followed by orthogonal x‐ray imaging using the integrated ExacTrac subsystem. Prior to delivery, the CM is built based on 20–40 s orthogonal fluoroscopy acquiring around 30 paired images at 1–3 fps, depending on current breathing motion, synchronously with IR tracking (at 60 fps). From the sequence, an orthogonal image pair is selected and the target manually localized by 2D–2D registration with template (planning) DRRs using the GTV to mask out all irrelevant regions. ExacTrac, then, iteratively localizes the target over consecutive frames. The CM is parameterized in the form of (detected) target positions (from orthogonal x‐ray sequence), as a function of IR marker position and velocity. Tumor detection has to be successful in at least 70% of frames in the sequence to be able to proceed with MLDTT treatment.^21^ During treatment, the external IR signal and CM combined reposition the irradiation field in response to tumor motion by means of pan and tilt rotations of the gimbaled MV x‐ray head.

At treatment, the validity of the CM is verified in sparse (1 fps) orthogonal x‐ray images. Target positions are automatically determined, compared to the CM, and deviations are displayed to the operator. Since the actual tumor shape in orthogonal projections is unknown for the different gantry positions, it is recommended to acquire *detection templates* (short fluoroscopy sequences followed by manual registration) from every gantry position since otherwise no deviation between the detected and predicted target position can be determined.^21^ Moreover, due to possible inter‐fraction changes in tumor shape and relative position of organs nearby, the acquisition of *detection templates* has to be performed on a per‐fraction basis. Routinely, however, *detection templates* are not acquired for each treatment due to increased time demands, patient exposure, and some gantry orientations being occluded by dense anatomic structures in the imaging direction. Checking the validity of the CM rests on the operators’ (consisting of at least one RTT and an experienced medical physicist) visual inspection of sparse orthogonal x‐ray images since ExacTrac is no longer able to determine deviations between the actual and predicted tumor position, hindering automatic beam interrupts, in case deviations occur.^21^

### 2.3 Data acquisition

The Vero4DRT is equipped with an on‐board amorphous silicon EPID mounted in‐line with the therapeutic beam. The detector has a source‐detector‐distance of 221.2 cm, 1024 × 1024 pixels matrix size, and 0.4 × 0.4 mm^2^ pixel spacing.^10^ Since the imager is orthogonal to the beam axis, rotations of the gimbaled x‐ray head in the pan and tilt directions correspond to the *x* (lateral) and *y* (longitudinal) axes in the imager plane. During MLDTT treatment of the 10 patients, BEV projections were captured at ∼3 fps with the EPID and streamed to a research workstation. MV sequences ranged between 24 and 172 frames. A total of 602 sequences, 34 070 frames were recorded over 85 fractions. An overview of the patient cohort, lesions characteristics, fields, fractions, and data analyzed is given in Table 1.

### 2.4 MV tracking algorithm

MV sequences were processed off‐line using the soft‐tissue feature tracker (SoFT).^31^ The algorithm requires a single gantry/ring angle‐specific frame for initialization. A detailed description can be found in previous work.^31^ Successful initialization results in the assignment of a set of feature landmarks (FLs), each of which is associated with a set of feature's attributes representative of the tracked target, defining the training image (*I*_train_). To determine the 2D target (centroid) position, $p^{\left(TAR\right)}\left(t\right)$, the algorithm (densely) samples each consecutive frame, referred to as query frame (*I*_query_), determines features’ descriptors and seeks the correspondence of the training set pair‐wise using features’ attributes.^31^ An extension to the previously reported application^31^ is its utility to segment the dynamic field aperture's (centroid) position, $p^{\left(APER\right)}\left(t\right)$, automatically, which forms the basis for prospective MV tracking simulations (Section 2.5).

### 2.5 MV verification, simulation strategy

The EPID emulates an independent camera, capturing both the target and field aperture. For instance, when the CM and tumor baseline systematically deviate (owing to transient changes in internal/external correlation), the gimbaled x‐ray head would not aim at the actual target accurately (i.e., geometric miss) perceived in the BEV. We simulated a method for independent intrafraction monitoring aiming to detect alignment errors beyond a predefined (3 mm) threshold. The approach assumes that following initial patient setup and CM acquisition, the spatial–temporal relationship between tumor and field aperture has been established, therefore their geometric relationship is expected to remain constant or within tolerance bounds. The MV tracking algorithm was fed with frame sequences as if coming from the frame grabber in real‐time.

**FIGURE 1:**
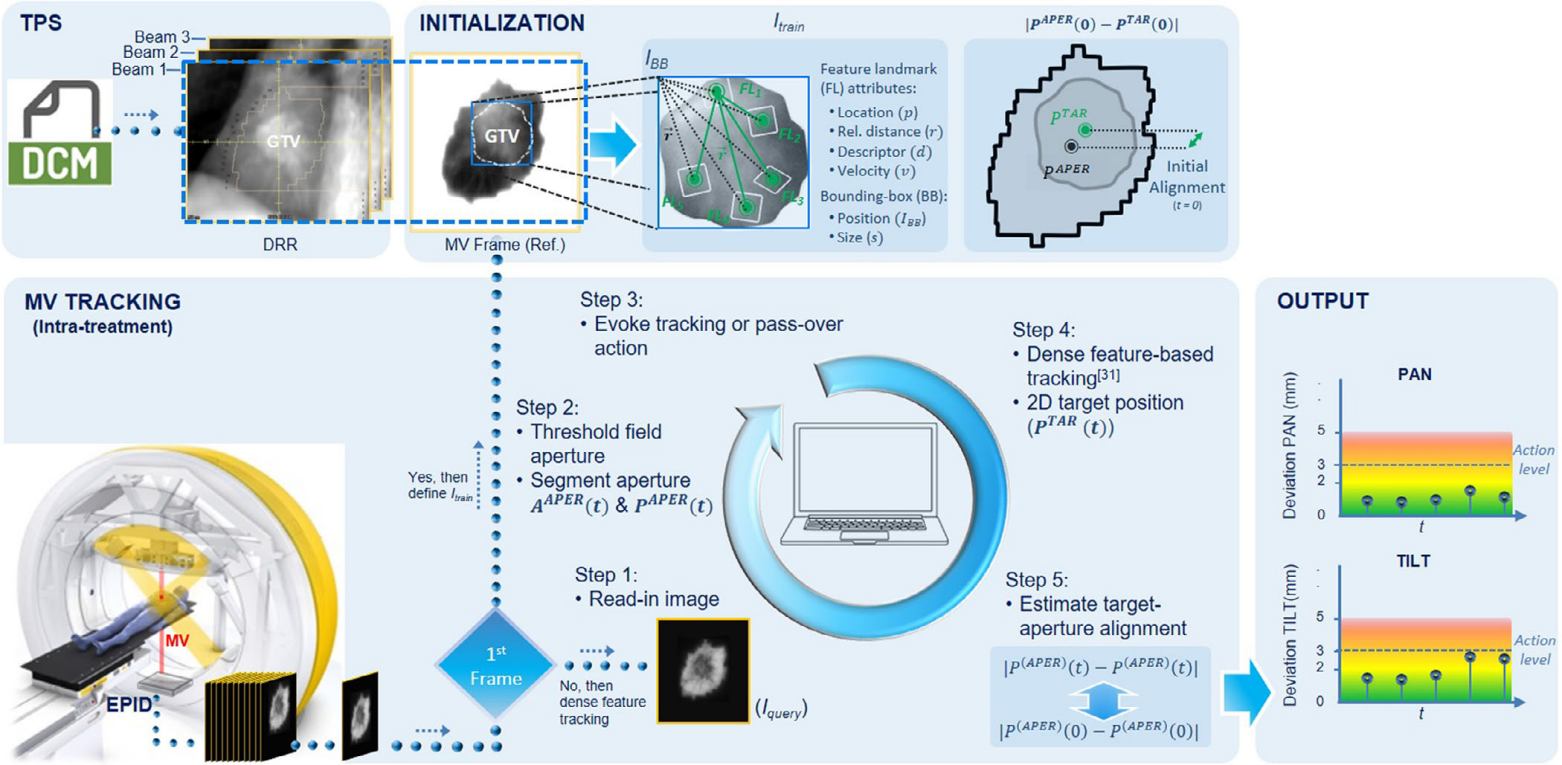
Schematic representation of the proposed MV‐based independent verification framework. DRRs (from treatment planning system [TPS]) aid GTV identification on initial MV frames at initialization of the MV tracking algorithm.

An overview of the methodology is outlined in Figure 1. The processing pipeline begins with the initialization of *I*_train_ and the reference (expected) geometry. By browsing each sequence, a representative frame in a corresponding phase where tumor visibility appeared the most optimal (e.g., EE) was selected. An important requirement is that the entire macroscopic tumor should be visible.^31^ To this end, a frame in an arbitrary port (field‐in‐field 3DCRT) or CP (step‐and‐shoot IMRT), in which the entire tumor mass was identified, was selected. Note that this mimics an idealized scenario maximizing target visibility, which is advantageous when trying to discern the target undergoing occlusions.^31^ In real clinical practice, a single open‐portal could be acquired by delivering a few MUs prior to treatment. On the reference frame, the region enclosing the projected macroscopic tumor was manually delineated. To aid tumor identification, digitally reconstructed radiographs (DRR) overlaid with the macroscopic tumor contour are displayed with reference frame side‐by‐side. The initial target, $p^{\left(TAR\right)}\left(t=0\right)$, aperture, $p^{\left(APER\right)}\left(t=0\right)$, and centroid positions are extracted and relative distances computed. During intra‐treatment monitoring, the algorithm takes as input each in‐coming frame (step 1) and the actual aperture area, $A^{\left(APER\right)}\left(t\right)$, is segmented (step 2). A minimum aperture size criterion is used to evoke an automatic MV tracking or pass‐over action (step 3). If the frame does not meet the criterion processing is terminated (pass‐over action) and returns to step 1, otherwise continues the MV tracking pipeline (step 4). The instantaneous $p^{\left(TAR\right)}\left(t\right)$ and $p^{\left(APER\right)}\left(t\right)$ are extracted, their relative distances computed, and compared to the reference geometry (step 5). The output is the in‐plane (pan/tilt) positional uncertainty of MLDTT treatment.

### 2.6 Analysis

#### 2.6.1 Tumor visibility dependency

Each MV sequence was retrospectively inspected on a per‐frame basis. Tumor visibility was ranked as poor, fair, or good depending on the level of tumor salient features visually identified by the observer. A good visibility ranking was assigned to sequences in which salient features could be identified over all frames for manual tracking, whereas a poor visibility score indicates that no traceable features could be identified. A fair visibility was assigned to those sequences in which the tumor shape was entirely or partially identifiable in some, but not all frames. This analysis included sequences acquired every second fraction only. We investigated the effect of subjective tumor visibility on MV tracking error.

#### 2.6.2 MV tracking accuracy

Since the ground truth location of the macroscopic tumor is unknown, manual tracking was also performed on each sequence. Similarly, the region enclosing the tumor mass (Section 2.4) was selected. Then, its position, $p^{\left(m\right)}\left(t\right)$, on consecutive frames was identified by manual shifts applied over the current frame. Manual tracking was repeated three times on each sequence. The order of frames was randomized to prevent any possible bias. The average of $p^{\left(m\right)}\left(t\right)$ for the three manually determined traces ($\left\langle p^{\left(m\right)}\right\rangle \left(t\right)$) was taken as an estimate of the true tumor trajectory. All coordinates were resolved in the imager plane, where the *x* and *y* directions correspond to pan and tilt rotation directions of the gimbaled x‐ray head, and scaled to the isocenter. To describe the accuracy of MV tracking, the point‐wise difference was calculated using:

(1) $$E_{SoFT}=\left|p^{\left(TAR\right)}\left(t\right)-\langle p^{\left(m\right)}\rangle \left(t\right)\right|$$

For each sequence, the mean, root‐mean‐square error (rmse), and 90th percentile were quantified. MV tracking success denotes the percentage of fields producing an output, regardless of the accuracy. To characterize the accuracy of MV tracking, we adopted a scheme comprising three performance levels as follows: good, fair, and poor. A “good” tracking performance was assigned to $E_{\mathrm{SoFT}}$ ≤ 2 mm, which is within the consensus of the expected errors along the imaging axis based on 2D imaging.^25^ “Fair” performance was assigned to $E_{\mathrm{SoFT}}$ ≤ 3 mm, while “poor” to $E_{\mathrm{SoFT}}$ > 3 mm. To estimate the error in manual tracking, a subset of 10 sequences with good visibility ratings were selected, and manual tracking was performed by three observers (one medical physicist [M.S.] and two radiation oncologists [S.K.B.S., A.R.]). The mean of the absolute difference between the three (centroid) trajectories was determined.

#### 2.6.3 Anatomy obstruction

Besides the low image quality and reduced field‐of‐view of BEV projections, target visibility may be obstructed by anatomic structures nearby, interfering with tumor localization. These may cause the clamping of tracked features to static structures (e.g., spine). On the other hand, the contraction/relaxation of the diaphragm and thorax expansion during respiration leads to opposing movements of the lung and ribs/chest wall. To assess the impact of anatomy‐related obstruction on MV tracking, sequences were inspected by the observer and classified into two subgroups: sequences corresponding to fields with and without obstructing anatomy identified in the BEV (Table 1), and resulting MV tracking errors compared.

#### 2.6.4 MLC occlusion

**FIGURE 2:**
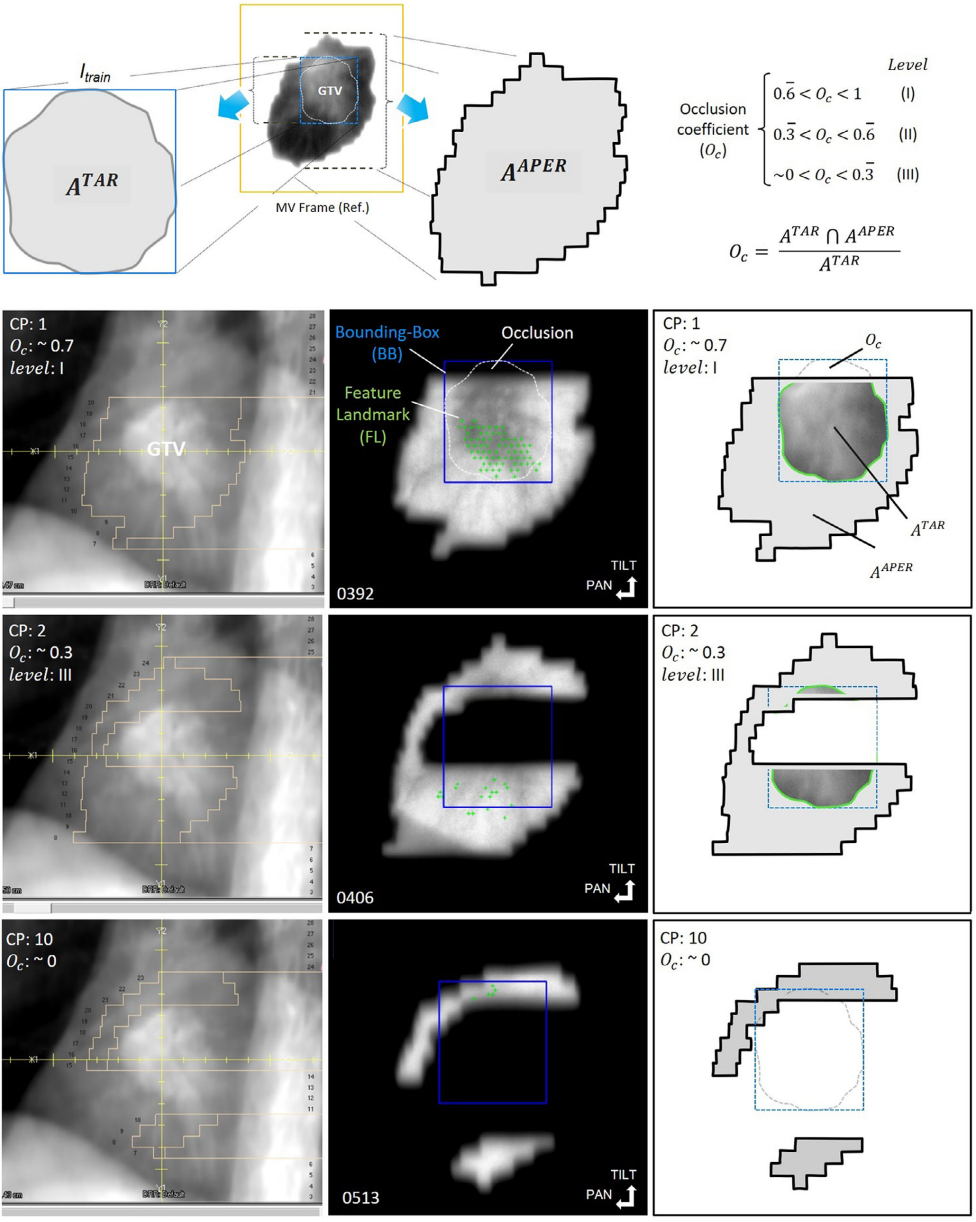
Estimation of occlusion coefficient ($O_{c}$) for a step‐and‐shoot IMRT field and comprising control point (CP) 1, 2, and 10. Calculating the area overlap between GTV (encompassed by region delineated during train frame [*I*_train_] initialization) and MLC aperture contour, occlusion was determined (range: 1–0), and classified into levels I–IV: slight, moderate, high, or total, accordingly. For each CP, the DRR with overlaid MLC aperture (left) and an arbitrary output frame (middle) is shown. The (green) cross marks represent the filtered set of FLs, whereas the (blue) bounding‐box represents the resultant estimated tumor position in the imager plane.

MLC occlusion is a challenging problem for MV‐based tracking methods. Especially, during IMRT delivery, the tracked target may become partially or completely occluded at times. To investigate the impact of MLC occlusion on the MV tracking error, sequences were classified into following two subgroups: (i) sequences corresponding to unmodulated fields, and (ii) sequences corresponding to fields impaired by MLC occlusion inherent to the planning process, for example, a sub‐field (SF) in field‐in‐field 3DCRT or an IMRT field comprised of various control points (CPs) in step‐and‐shoot IMRT. For the latter subgroup, occlusions were further categorized into level I (slight), II (moderate), III (high), or IV (total), depending on the amount of gross tumor volume (GTV) projection occlusion incurred by the MLC. An occlusion coefficient ($O_{c}$), a measure of unoccluded‐to‐occluded ratio of the GTV in the BEV, was determined. For each SF‐ and CP‐specific sequence, $O_{c}$ was determined by calculating the area overlap between GTV projection and MLC aperture:

(2) $$O_{c}=\frac{A^{\mathrm{TAR}}\cap A^{\mathrm{APER}}}{A^{\mathrm{TAR}}}$$

where $A^{\mathrm{TAR}}$ is the area contour enclosing the tracked target (Section 2.4) and $A^{\mathrm{APER}}$ is the area of the contour defined by MLC aperture on the reference frame used during MV tracking initialization (Figure 1). An $O_{c}$ = 0 signifies no spatial overlap between GTV and MLC contours (i.e., GTV fully occluded by MLC), whereas $O_{c}$ = 1 indicates complete overlap (i.e., GTV fully unoccluded). We associated level I to coefficient values (0.6 < $O_{c}$ < 1), level II (0.3 < $O_{c}$ < 0.6), and level III (0 < $O_{c}$ < 0.3), respectively. An illustration of $O_{c}$ estimation for a representative IMRT field (case 32b, field C6) is shown in Figure 2. At the top, the region delineated for *I*_train_ initialization is shown. From this region, $A^{\mathrm{TAR}}$ is determined. Using Equation (2), $O_{c}$ values for three representative CPs (CP1, 2, and 10) are shown in row 2, 3, and 4. For each CP, the DRR with GTV and overlaid MLC aperture (left), denotative of the expected occlusion, whereas the estimated $O_{c}$ values (right) are displayed. An arbitrary output frame, from which $A^{\mathrm{APER}}$ is estimated, can also be observed (middle). The green cross marks in the MV frame represent the filtered set of feature landmarks, while the blue bounding‐box's centroid indicates the actual target position in the pan and tilt direction of the gimbaled x‐ray head. Inspecting DRRs and projections, it is apparent that the GTV is occluded by the MLC, at times. Since multiple FLs are simultaneously tracked, the algorithm is capable to resolve the tumor and estimate its position, overcoming occlusions. In scenarios where the aperture area falls below a pre‐defined value (area < 16 × 16 pixels [FL's descriptor size] × 10 [minimum number of FLs]),^31^ MV tracking is discontinued or resumed otherwise.

#### 2.6.5 MLDTT geometric uncertainty

For each frame, the target‐aperture alignment error was determined as described in Section 2.5. If the alignment error on the current frame is <3 mm, it is considered to be within tolerance, or beyond otherwise. To this end, first, the tracked (target) location was determined as correctly tracked if $p^{\left(TAR\right)}\left(t\right)$ was within 2 mm accuracy (relative to its ground truth position ($\left\langle p^{\left(m\right)}\right\rangle \left(t\right)$) described in Section 2.6.2), defining a *true positive*. Conversely, a tracking event failing the criterion was defined as a *false positive* (i.e., an incorrectly tracked position). The efficacy of MV tracking in intrafraction monitoring of MLDTT treatment was described by the true positive rate (TPR):

(3) $$\mathrm{TPR}=\frac{\mathrm{true}\;\mathrm{positives}}{\mathrm{true}\;\mathrm{positives}+\mathrm{false}\;\mathrm{positives}}$$

We analyzed the percentage of time the alignment error exceeded the 3 mm threshold, which served as a measure to quantify (possible) overthreshold misalignments, albeit routine x‐ray verification, during treatment. We also determined the percentage of time the alignment was within the planned GTV‐PTV margin (e.g., 5 mm in this study) and exceeding the 5, 7, and 10 mm uncertainty ranges. To estimate the geometric uncertainty of MLDTT treatment, $E_{\mathrm{MLDTT}}$, the root‐mean‐square error between $p^{\left(\mathrm{TAR}\right)}\left(t\right)$ and $p^{\left(\mathrm{APER}\right)}\left(t\right)$ for each sequence was determined using:

(4) $$E_{\mathrm{MLDTT}}=\sqrt{\frac{\sum_{t}^{N}{\left(p^{\left(\mathrm{APER}\right)}\left(t\right)-p^{\left(\mathrm{TAR}\right)}\left(t\right)\right)}^{2}}{N}}$$

where N is the number of frames in the sequence. For comparison, the geometric error based on the ground truth target trajectory was also estimated. The group mean (mean of mean errors), *M*; the systematic (standard deviation of mean errors) *Σ*; as well as the random (root‐mean‐square of individual standard deviations) *σ* errors were determined based on the van Herk's formalism.^32^

## 3 RESULTS

### 3.1 Demographic assessment

The median GTV volume and diameter were 3.4 cm^3^ (range: 1.5–24.8 cm^3^) and 1.9 cm (range: 1.2–6.8 cm). Lesions were evenly distributed between the left and right lungs. The median p–p motion amplitude was 10.7 mm (range: 3.1–45 mm). Overall, tumors located in the lower lung lobes showed higher (center of mass) p–p motion amplitude (median: 13.9 mm) compared to those located in the upper lobes (median: 4.6 mm). Of the planned 73 gantry‐ring angle combinations anatomy‐related target obstruction was identified in 52/73 (71.2%), while MLC‐related occlusion, inherent to field‐in‐field 3DCRT and IMRT planning, in 25/73 (34%), respectively. A total of 7 SFs and 84 CPs (91 all combined) were identified (Table 1).

### 3.2 Interobserver error of manual tracking

The mean absolute difference between manual centroid trajectories was 0.5 ± 0.1 and 0.6 ± 0.2 mm in the pan and tilt directions, with maximum deviations up to 3.8 and 4.3 mm in pan and tilt directions, respectively. Due to the small interobserver uncertainty, the following in‐depth analysis was based on the manual tracking of one observer (M.S.) who has 10 years of experience identifying and analyzing lung tumors in MV images.

### 3.3 Tumor visibility dependency

**FIGURE 3:**
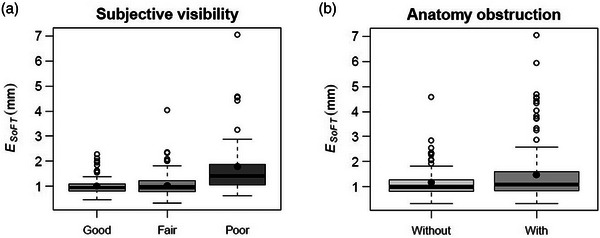
(a) Boxplots showing the relationship between MV tracking errors ($E_{\mathrm{SoFT}}$) and the subjective tumor visibility, and (b) anatomy‐related obstruction.

Subjective visibility assessment revealed a relationship between MV tracking error and tumor visibility. Of the evaluated sequences, 25.4%, 38%, and 36.6% were identified to exhibit poor, fair, and good tumor visibility. The mean $E_{\mathrm{SoFT}}$ was 1.8 ± 1.2 mm (range: 0.6–7.0 mm), 1.1 ± 0.5 mm (range: 0.3–4.1 mm), and 1.0 ± 0.4 mm (range: 0.5–2.3 mm) for the poor, fair, and good visibility subgroups, respectively (Figure 3a).

### 3.4 Anatomy‐related obstruction

**FIGURE 4:**
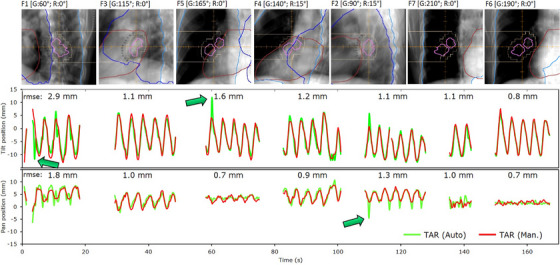
Tumor centroid positions for automatic (green) and manual (red) MV tracking for patient 70, fraction 1 comprised of eight gantry‐ring angle combinations (beams F1‐7). For each beam, DRRs, gantry‐ring angles with overlaid overlapping anatomy (heart, spine, ribs) and GTVs (two macroscopic tumors highlighted in pink) are shown (top). The $E_{\mathrm{SoFT}}$ is shown above each field trace for both the pan and tilt directions (bottom).

The mean $E_{\mathrm{SoFT}}$ for sequences corresponding to fields traversing neighboring structures (most commonly heart, spine, and ribs) was 1.4 ± 0.9 mm (range: 0.3–7.0 mm), whereas for those with no obstruction was 1.1 ± 0.6 mm (range: 0.3–4.6 mm). Obstruction led to increased errors in MV tracking (Figure 3b). An example (case 70) of overlapping anatomy obstruction is illustrated in Figure 4. DRRs and corresponding gantry‐ring angles have been added to highlight the overlapping spine and heart. The extracted trajectories agreed closely with the ground truth. The highest error was observed for field F1 [G:60°, R:0°] with $E_{\mathrm{SoFT}}$ values of 1.8 and 2.9 mm in the pan and tilt direction, and a maximum (single) deviation of ∼13.7 mm (tilt) at *t* = 3.9 s (green arrows), attributed to pinning of FLs to the overlapping (static) spine. High $E_{\mathrm{SoFT}}$ values were also observed for field traces F5 and F2 obstructed by heart and shoulder bones with $E_{\mathrm{SoFT}}$ < 1.6 mm, although anomalous single deviations up to ∼8.3 (*t* = ∼60 s) and 4.9 mm (*t* = ∼110 s) were recorded (Figure 4). Nevertheless, even under these challenging scenarios, the algorithm detected the tracked target, handling the presence of overlapping static/dynamic structures, and determined its trajectory satisfactorily.

### 3.5 MLC occlusion

**FIGURE 5:**
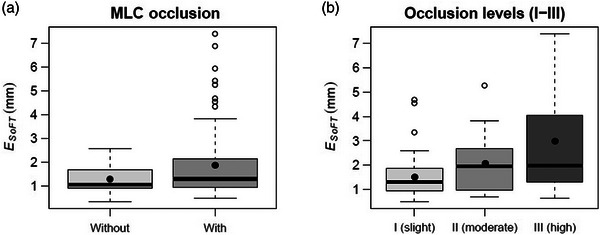
(a) Errors in MV tracking for sequences with and without identified (by the observer) MLC‐related occlusions, and (b) occlusion levels I–III. The solid circles and horizontal lines inside each boxplot represent the mean and median. The lower‐ and upper‐whiskers denote the minimum and maximum of the dataset, whereas the points beyond (whisker) outliers.

Subjective occlusion levels I, II, and III were identified in 17.6%, 15.4%, and 16.5% of corresponding sequences (Table 1). Total target occlusions were identified in 25.3% of SFs/CPs, and thus, rendered unsuitable for MV tracking. Examples of levels I, III, and IV corresponding to CPs 1, 2, and 10, respectively, are illustrated in Figure 2. Overall, MV tracking was successful for 56/91 (61.5%) of the SFs/CPs (Table 2). Across the analyzed sequences, the mean $E_{\mathrm{SoFT}}$ for the pan/tilt combined was 1.8 ± 1.5 mm for the subgroup with the target undergoing occlusions, compared to 1.3 ± 0.5 mm for the subgroup without (Figure 5a). As expected, the accuracy was dependent on the unoccluded‐to‐occluded ratio of GTV projection visible in the BEV. Slight occlusions were handled with ease, while moderate and high occlusions posed more stringent challenges to complete loss of MV tracking information. The mean $E_{\mathrm{SoFT}}$ for sequences with identified occlusions levels I–III was 1.6 ± 1.0 mm (level I), 2.4 ± 1.3 mm (level II), and 2.8 ± 2.0 mm (level III), respectively (Figure 5b). The interquartile range of error distributions was 0.9 mm (level I), 1.6 mm (level II), and 2.6 mm (level III), whereas the 90th percentile was 3.0, 3.7, and 6.1 mm, respectively.

**TABLE 2:** MV tracking success, performance rate, and measured tracking accuracy of absolute differences between manual and automatic MV tracking (mean, root‐mean‐square‐error, and 90th percentile in mm) for both pan and tilt directions.

	Fields (%)		Frames (%)				Pan						Tilt					
Case	Main	SF|CP	SF|CP				|$\left\langle p^{\left(m\right)}\right\rangle$—$p^{\left(SoFT\right)}$|			Performance (%)			|$\left\langle p^{\left(m\right)}\right\rangle$—$p^{\left(SoFT\right)}$|			Peformance (%)		
					
No.	Succ.	Succ.	Succ.	Succ.^b^	Fail	Excl.^a^	Mean	RMSE	90th	Good	Fair	Poor	Mean	RMSE	90th	Good	Fair	Poor
30	100		–	–		–	0.9	1.3	4.7	91.3	5.9	2.8	1.0	1.3	5.7	90.5	7.3	2.2
32	–	84.6	65.4	80.9	15.4	19.1	1.1	1.4	3.2	83.5	11.8	4.6	1.6	2.0	5.1	75.4	12.9	11.6
	–	63.3	69.3	88.5	9.0	21.7												
34	83.3	33.3	28.9	100	–	66.7	0.7	0.9	2.5	97.0	3.0	0.0	0.7	0.9	1.7	97.8	2.2	0.0
42	75.0	–	–	–		–	0.6	0.8	1.7	97.2	2.8	0.0	0.5	0.6	1.2	98.6	0.7	0.7
43	85.7	0.0	0.0	–	100	–	1.0	1.3	5.2	88.3	9.2	2.5	2.3	3.5	14.4	60.8	15.8	23.3
44	–	54.5	69.6	85.8	12.0	18.9	1.5	1.7	3.4	87.8	6.6	5.6	1.4	1.6	3.9	82.2	10.0	7.9
45	100	0.0	0.0	–	100	–	0.5	0.6	1.7	95.2	4.2	0.6	0.8	0.6	1.2	94.6	5.4	0.0
51	87.5	0.0	–	–		100	0.8	1.0	1.8	96.5	3.2	0.3	1.0	1.3	2.4	88.3	9.2	266
64	85.7	–	–	–		–	0.8	1.0	2.1	93.6	4.2	2.2	0.7	1.0	1.9	95.2	3.5	1.3
70	100	100	100	–	0.0	–	0.9	1.3	2.2	93.1	4.5	2.3	1.1	1.6	2.6	86.8	9.6	3.7
	89.7	61.5	55.5	88.8	27.3	32.7	0.9	1.2	2.9	92.4	5.5	2.1	1.1	1.4	4.0	87.0	7.7	5.3

^a^ Total number of frames (in percentage) excluded due to total target occlusions by MLC.

^b^ Success rate after exclusion of sequences exhibiting complete target occlusion by MLC.

An example of MV tracking and occlusion handling during IMRT delivery can be visualized in Supporting Information S1. Since sufficient FLs are tracked across the target, the algorithm is capable to detect the tumor and estimate its position (indicated by the blue bounding‐box). After excluding CPs with complete tumor occlusion (e.g., CP10 and 11), results revealed a relative high success rate. It is noteworthy to emphasize that the sequence comprises periods of beam‐ON/‐OFF, which correspond to transitions of the MLC between CPs for step‐and‐shoot IMRT delivery. Since the MV imager operates in cine mode, frames are continuously read out irrespective of the beam‐on/off status. This explains periods of complete dark (beam‐OFF), or quasi dark frames (attributed to trapped charge in the imager, e.g., frames 0414, 0419–0420, 0439–0440, 0460–0463), at times. Even in the presence of frames with low signal, the MV tracking algorithm tries to localize the tracked target.

### 3.6 MV tracking success and performance

Table 2 summarizes the success, performance, and measured accuracy of MV tracking for all 10 patients. Automatic tumor detection was successful for 49/55 (89.7%) of (unmodulated) main fields in 3DCRT. Unsuccessful MV tracking mostly occurred when image contrast was dominated by overlapping structures of high attenuation obscuring the tumor. As for modulated fields, MV tracking was successful for 56/68 (82.4%) of CPs/SFs (excluding 23 CPs/SFs exhibiting complete GTV occlusion). The success rate, defining the percentage of the frames where MV tracking was possible, was 55.5% with most failures arising from occlusions of the GTV by MLCs. MV tracking failed in 27.3% of the frames, with the majority of incidences where the tracking signal was lost only for a single image, and a maximum period of three consecutive frames. When excluding frames exhibiting total occlusions by MLCs (32.7%), the tracking success rate yielded 88.8% (Table 2).

**FIGURE 6:**
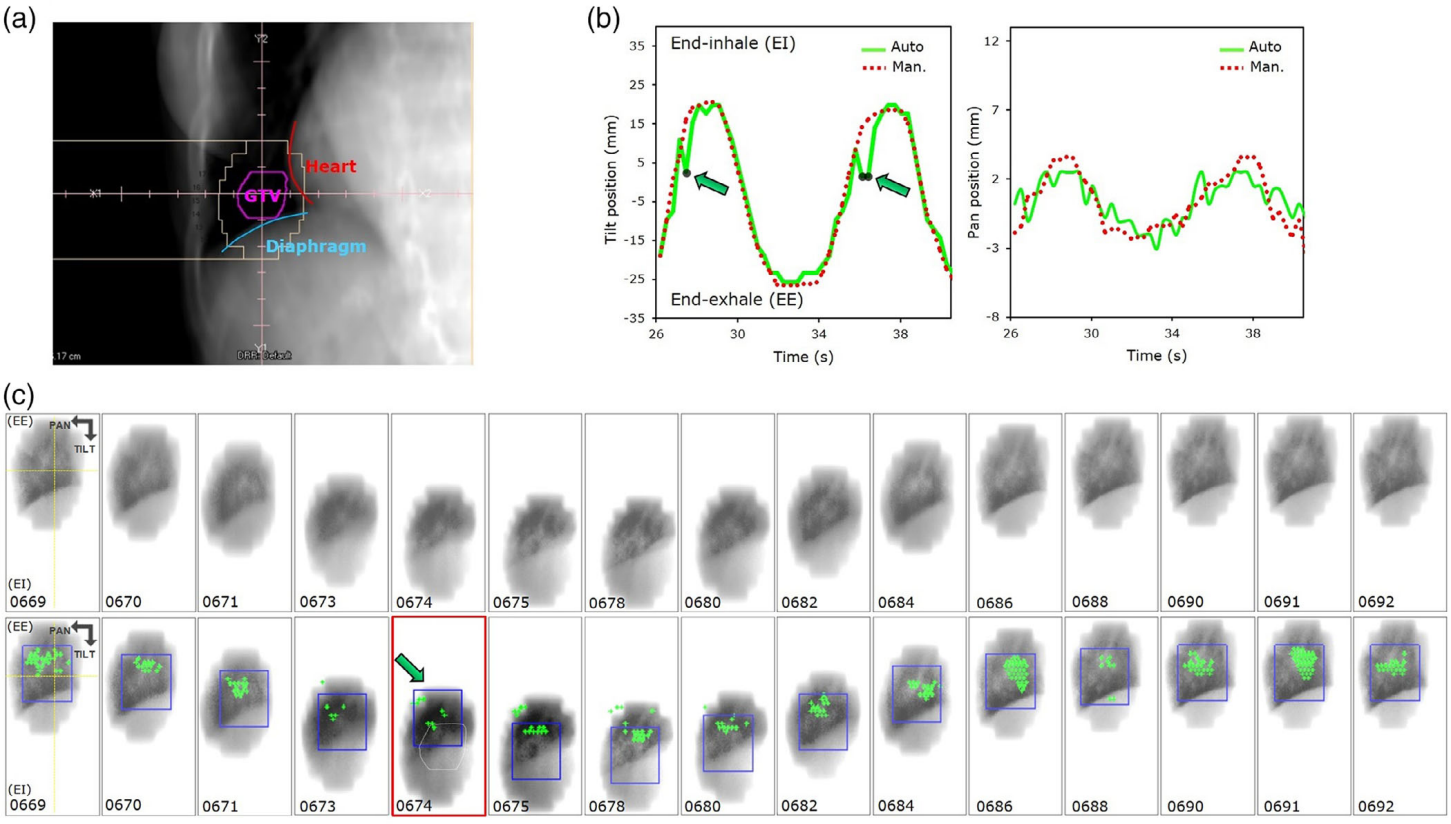
Comparison of automatic versus manual tracking corresponding to patient 43 (field 2). (a) DRR with overlaid GTV and overlapping heart and diaphragm. (b) MV tracking output (green) and reference (dotted red) trajectories spanning over two breathing cycles. (c) Pre‐processed (contrast‐enhanced) frames (top row) and algorithm output with filtered FLs (bottom row). The bounding‐box represents the detected tumor on the current frame.

Averaged across all cases, the $E_{\mathrm{SoFT}}$ was 1.2 ± 0.3 mm in the pan and 1.4 ± 0.8 mm in the tilt direction, equivalent to a 2D (pan/tilt combined in quadrature) accuracy of 1.8 mm. The mean (absolute) error and 90th percentile are also given. The MV tracking performance with good, fair, and poor accuracy was 92.1%, 5.5%, and 2.3% for pan, and 86.6%, 7.6%, and 5.7% for tilt, respectively (Table 2). The MV tracking accuracy and performance in the tilt direction are slightly dependent on the motion magnitude. For all cases, the mean $E_{\mathrm{SoFT}}$ was <2 mm except for case 43, which showed the highest error (3.5 mm) and lowest “good” performance (60.8%) in the tilt direction. Similarly, cases 32 and 44 (IMRT deliveries) yielded reduced accuracy (2.0 and 1.6 mm) and “good” performance (75.4% and 82.2%). An example of MV tracking output for representative case 43 (field A2) under stringent conditions, namely reduced tumor contrast, tumor/surrounding tissue deformation, obstruction (e.g., diaphragm), and significant (∼4.5 cm) p–p motion amplitude is illustrated in Figure 6. Both the train and query frames were sampled using a grid size 9 × 9 pixels and 120 FLs across the tumor were tracked at ∼86 ms/frame (processing time) overlaid with GTV and overlapping diaphragm and heart is shown at the top‐left (Figure 6a). The two rows of sequences, spanning over approx. one breathing cycle, represent the (contrast‐enhanced) pre‐processed (top) and output frames (bottom). The green cross marks (sequence at the bottom) represent the filtered FLs, whereas the blue bounding‐box shows the resolved tumor position along the pan and tilt motion directions. The extracted internal maps (green) compared to the manual ground‐truth (dotted red) for the subinterval (*t* = 26–39 s) are shown at the top right (Figure 6b). Generally, the trajectory resolved by MV tracking closely resembled that determined by manual tracking. The green arrows highlight the occurrence of single (anomalous) deviations in tumor localization near the end‐inhale (frame 0674). These were attributed to considerable changes in tumor appearance due to tissue deformation, high heterogeneity of surrounding tissue, and diaphragm obstruction. Of note, the reconstructed trajectory is in the pan direction. The superimposed high‐frequency component is explained by cardiac‐induced motion. The MV tracking algorithm was capable to discern the motion attributed to heart beat. A video clip in the Supporting Information (S2) is available showing pronounced heart‐beat motion around end‐exhale (between frames 0688–0694 and 0717–723).

### 3.7 MLDTT geometric uncertainty

**FIGURE 7:**
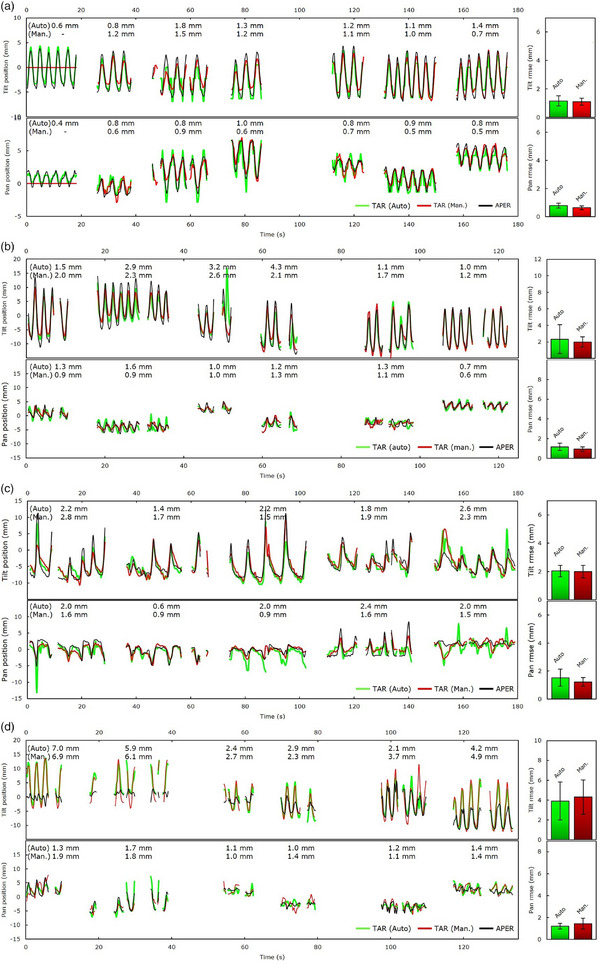
Application of MV intrafraction monitoring to four treatment fractions corresponding to cases 51 (fraction 1) (a), 32 (fraction 3) (b), 30 (fraction 2) (c), and 32 (fraction 1) (d). Position (center of mass) traces in the pan and tilt direction are plotted for the SoFT‐derived (auto) field aperture (gray) and tracked target (green), overlaid with the tumor manual ground truth (red). The bar charts to the right of each displacement map represent the fraction's mean $E_{\mathrm{MLDTT}}$ for auto‐tracking, compared to manual tracking.

In Figure 7, the output trajectories in pan and tilt directions for four treatment fractions are shown. For each case, the tumor (green) and dynamic field aperture (black) centroid position overlaid with the ground truth trajectory (red) are plotted. Each trajectory is relative to the initial frame used for the (MV tracking) algorithm initialization.^31^ The bar graphs on the right of each displacement map illustrate the fraction's mean $E_{\mathrm{MLDTT}}$ and the whiskers the standard deviation. Figure 7a,b portray MV tracking under (or quasi) regular motion corresponding to patients 51 (fraction 1) and 32 (fraction 3). The higher mean $E_{\mathrm{MLDTT}}$ in (b) is attributed to the more irregular and larger tumor motion amplitude of patient 32, compared to patient 51. Figure 7c represents a less regular motion with erratic changes in motion amplitude and period, which led to intermittent periods of beam‐on/‐off and intensity fluctuations in MV projection images. The MV tracking algorithm detected the target on visible frames, discontinued tracking upon obscured frames, and retrieved the target's position once beam‐on resumed. For all cases, the $E_{\mathrm{MLDTT}}$ based on automatic and manual tracking are comparable. This confirms not only the high predictive value of the Vero's CM, but also its capability to act upon patient‐specific changes in real‐time. However, exceptions occurred. Figure 7d shows the case with the highest $E_{\mathrm{MLDTT}}$ for the patient cohort (patient 32, fraction 1). The largest errors were found during the delivery of field 1 ($E_{\mathrm{MLDTT}}$ = 7.0 mm) and 2 ($E_{\mathrm{MLDTT}}$= 5.9 mm). These are manifested as misalignments in amplitude and phase between tumor (green) and aperture (black) trajectories, which were successfully detected and quantified by MV tracking. At about *t* = 55 s the internal/external correlation is re‐established, improving phase and amplitude agreement.

A summary of the TSR and MV‐measured geometric uncertainty of MLDTT treatment is listed in Table 3. Uninterrupted intrafraction position monitoring was possible 89.6% of the time for both the pan and tilt combined. The positional uncertainty beyond the 3 mm threshold was observed 5.6% (pan) and 10.4% (tilt) of the time. The target‐aperture alignment within the 5 mm GTV‐PTV margin was 97.9% and 96.7% for the pan and tilt, respectively. For cases 30, 32, and 43, alignment errors >5 mm were observed in 9.9% (pan), 15.8% and 12.6% (tilt) of the time. For case 30, the overthreshold incidences of 9.9% (pan) compared to 1% (tilt) are explained by the dominant left–right motion. Overall, the $E_{\mathrm{MLDTT}}$ determined by MV tracking was comparable to that of manual tracking. Averaged across all patients, the estimated $E_{\mathrm{MLDTT}}$ was 1.2 ± 0.6 mm (range: 0.5–2.6 mm) and 1.7 ± 0.9 mm (range: 0.5–3.5 mm) for the pan and tilt, respectively, compared to 1.1 ± 0.5 mm (range: 0.4–2.2 mm) for the pan and 1.6 ± 0.8 mm (range: 0.6–3.2 mm) for the tilt based‐on manual tracking. While the high $E_{\mathrm{MLDTT}}$ of case 43 can be attributed to the considerably larger tumor motion amplitude, that of case 32 due to truly increasing residual errors in target‐aperture alignment on a given day (e.g., Figure 7d); the percentage difference in (mean) $E_{\mathrm{MLDTT}}$ between auto‐ and manual tracking for the tilt is about 33.3% for case 43 and 3.2% for case 32 (Table 3). A summary of mean RMSE for the two scenarios, together with the population *M*, *Σ*, and *σ* is shown in Supporting Information S3. Using the van Herk formalism,^32^ the *Σ* and *σ* were 0.5 and 0.8 mm for pan, and 0.7 and 1.2 mm for tilt direction, respectively, compared to 0.3 and 0.5 mm for the pan, and 0.6 and 0.9 mm for the tilt based on the manual ground truth. Therefore, the margins needed to ensure a minimum target dose of 95% with 90% probability^32^ would be 1.8 mm in the pan and 2.5 mm in the tilt, which is similar to the ground truth estimates of 1.3 and 2.2 mm for the pan and tilt, respectively.

**TABLE 3:** True positive rate (TSR) of MV monitoring and geometric uncertainty of MLDTT treatment.

			% of displacement										*E_MLDTT_* (mm)			
Patient	TSR (%)		≤3 mm		≤5 mm		>5 mm		>7 mm		>10 mm		Auto		Manual	
No.	Pan	Tilt	Pan	Tilt	Pan	Tilt	Pan	Tilt	Pan	Tilt	Pan	Tilt	Pan	Tilt	Pan	Tilt
30	89.1	90.1	79.6	86.2	90.1	99.0	9.9	1.0	6.6	0.6	1.6	0.1	2.6	1.9	2.2	1.9
32	82.8	77.0	90.1	70.2	97.0	84.2	3.0	15.8	1.0	8.2	0.7	3.9	1.3	3.1	1.3	3.2
34	97.9	93.8	98.9	97.0	100.0	100.0	0.0	0.0	0.0	0.0	0.0	0.0	0.8	1.3	0.9	1.0
42	95.6	87.7	100.0	98.9	100.0	100.0	0.0	0.0	0.0	0.0	0.0	0.0	0.6	0.8	0.4	0.6
43	94.6	87.8	90.9	71.3	98.6	87.4	1.4	12.6	0.3	5.6	0.0	3.1	1.8	3.5	1.5	2.5
44	89.1	80.5	95.0	93.3	98.3	99.3	1.7	0.7	0.5	0.0	0.0	0.0	1.3	1.7	1.0	1.6
45	92.2	95.2	100.0	100.0	100.0	100.0	0.0	0.0	0.0	0.0	0.0	0.0	0.5	0.5	0.8	0.7
51	96.4	92.0	97.3	96.7	99.9	100.0	0.1	0.0	0.0	0.0	0.0	0.0	1.0	1.5	0.8	1.2
64	82.1	92.8	94.3	92.6	96.0	98.0	4.0	2.0	2.9	1.0	0.6	0.0	1.0	1.5	0.8	1.3
70	93.5	81.4	97.5	89.8	99.4	98.9	0.6	1.1	0.5	0.5	0.4	0.5	1.3	1.9	1.0	1.7
*Group*																
*mean*	91.3	87.8	94.4	89.6	97.9	96.7	2.1	3.3	1.2	1.6	0.3	0.8	1.2	1.7	1.1	1.6

*Note*: The *E*_MLDTT_ for both auto‐ and manual‐tracking values are reported in mm. The displacement percentages state the fraction of the beam‐on time with a tumor‐field aperture alignment error ≤3 and 5 mm GTV‐to‐PTV margins as well as exceeding 5, 7, and 10 mm.

## 4 DISCUSSION

The O‐ring gantry design of the Vero4DRT and of next‐generation closed‐bore linacs^33^ makes the in‐line flat panel detector appealing for on‐line intrafraction monitoring. MV tracking was successful for 89.4% of unmodulated (3DCRT) and 82.4% of modulated CPs (IMRT) and SFs (field‐in‐field 3DCRT) fields and gantry‐ring angle combinations. Previously, Richter et al.^27^ and Serpa et al.^34^ evaluated a single‐template algorithm and found that continuous position monitoring was possible in 47% of the fields for ITV‐based and 67% for gated‐based 3DCRT deliveries. Unlike our presented method, generous field apertures were used. Moreover, MLC occlusions, in the case of sub‐fields (field‐in‐field 3DCRT) deliveries or due to the proximity of GTV to the field boundary, were excluded or not addressed. Systematic changes in motion baseline causing the target to come into close proximity to the field edges led to increased errors or loss of tracking. In contrast, our proposed method yielded a higher success rate, handling partial occlusions at the field edges. Nonetheless, both studies led to the conceptual development of feature tracking and SoFT.^31^ Although this analysis was performed retrospectively and offline, the algorithm was tested in a real‐time scenario using data that would be accessible from any real‐time imaging system. The processing time ranged 86–320 ms per frame. The AAPM TG76 recommended a latency no longer than 500 ms.

The MV tracking accuracy was 1.2 ± 0.3 mm (pan) and 1.4 ± 0.8 mm (tilt) direction, yielding a resultant 2D accuracy of 1.8 mm, which is comparable to other related works.^26^, ^27^, ^28^, ^29^, ^30^ An additional uncertainty of 0.5 ± 0.1 mm (pan) and 0.6 ± 0.2 mm (tilt) should be added due to the interobserver error in manual tracking. The high accuracy may in part be explained by the eligibility criteria of patients undergoing MLDTT, namely localized lesions with sufficient visibility in kV fluoroscopy. The subjective visibility test, however, revealed that “good” tumor visibility comprised 36.6% of the sequences only. Moreover, this study included qualitative clinical data reflecting different tumor locations, sizes, and motion magnitudes. The smallest lesion the algorithm tracked was case 30 (volume: 1.5 cm^3^; diameter: 1.2 cm) and yielded a “good” performance 90.9% (pan/tilt combined) of the time. Overall, 6/10 cases had GTVs with diameters <1.9 cm. Case 43 (GTV volume: 3.7 cm^3^; p–p amplitude: 45 mm) constituted another example of MV tracking under stringent conditions, namely significant motion amplitude/speed, coupled with increased image blurring (Figure 6). This highlights the pivotal role of image quality in markerless MV tracking. Optimizing imaging acquisition settings, for example, increasing acquisition frame rate, may reduce motion blurring between frames. However, the handling of changes in tumor appearance rests on the algorithm's (dense) multi‐feature tracking characteristic and the rotation‐/scale‐invariance properties of SIFT descriptors.^31^

MV tracking performance is dependent on patient's traversed anatomy, and hence on tumor location. Overlying structures with high attenuation, for example, the vertebrae, reduce the saliency of feature landmarks and thus MV tracking accuracy. Clamping of candidate features to static structures, for instance, field F1 in Figure 4, was associated with increased MV tracking errors ($E_{\mathrm{SoFT}}$ = 2.4 mm, maximum [single] deviation up to ∼13.7 mm). These were handled by the algorithm's feature symmetry filtering that maintains spatial coherence (candidate landmarks that move in unison), although false matches remained at times. Optimal symmetry thresholds were adjusted empirically. Numerous studies have highlighted the impact of overlying anatomy and gantry angle dependency on tracking accuracy for MV‐based^28^, ^29^, ^30^ as well as kV‐based methods.^35^, ^36^, ^37^ Only Yip et al. quantified the effect of a patient's traversed anatomy on MV tracking, proposing ranges of gantry‐couch angle combinations for improved tumor visibility.^30^ However, these might not always be clinically practicable. Dual energy (DE) radiography may be beneficial in facilitating bone‐free images and improved tumor visibility. However, denser soft‐tissue structures (e.g., heart and diaphragm) would still pose challenges for tumors in their vicinity. Three cases (32, 43, and 44) comprised tumors located near the diaphragm. Among these, patient 43 showed the lowest good performance. Besides its small volume (3.7 cm^3^), the lesion exhibited poor visibility. The higher MV tracking uncertainty may also be attributed to its low density.^37^

MLC occlusion results in the loss of traceable features or total occlusions at times. MV tracking was not possible in 25.3% of CPs/SFs and 32.7% of the frames due to total occlusions. At present, a minimum aperture size criterion is used to evoke an automatic tracking or pass‐over action. This reduces the MV tracking efficiency (number of successfully tracked frames out of the total). In essence, inferences from TPS may be utilized to indicate expected periods of high/total occlusions. Moreover, lung tumors exhibit certain periodic characteristics that could be effectively learned and incorporated into a model to fill the blindspots (while the tumor is being fully occluded). Recently, Ferguson et al.^38^ demonstrated the feasibility of the multi‐region algorithm^29^ (extended implementation) for markerless MV tracking during VMAT delivery. Our approach differs from Ferguson et al. in that it operates on the lowest level of abstraction (features) instead of on a pixel basis via template matching. The authors reported a tracking uncertainty of 1.3 mm, although the method was tested on a limited clinical dataset (one patient, nine fields, motion magnitude ∼8 mm). In our presented study, the mean $E_{\mathrm{SoFT}}$ for the subgroup with MLC‐related occlusion was 1.8 mm (IQR = 1.1 mm; 90th percentile = 4.7 mm), including tumors with larger motion magnitudes (mean: 10.7 mm; range: 3.1–45 mm). Nevertheless, Ferguson et al. represents a potential extension of our presented method, that is, MV tracking during rotational delivery.

The percentage of time the alignment exceeded the 3 mm threshold, which quantified the residual targeting uncertainty in clinical MLDTT treatment, was 5.6% (pan) and 10.4% (tilt). Previously, Depuydt et al.^11^ evaluated the geometric uncertainty of (marker‐based) DTT treatment based on x‐ray imaging. They reported the geometric coverage within 5 mm to be 97.6% on average. In our study, the MV‐estimated $E_{\mathrm{MLDTT}}$ was within 5 mm for 97.3% (pan/tilt) of the time. However, when considering the inter‐patient variability the 5 mm GTV‐PTV coverage below 95% was observed for patients 30, 32, and 43. These were mainly attributed to patient‐/fraction‐specific irregularities in breathing. Figure 7d is a representative example of increased positional errors, albeit routine x‐ray verification, during MLDTT treatment that would have been caught by MV tracking. To restate, the Vero4DRT is equipped with orthogonal x‐ray imaging which combined with feedback from (gimbal's) positional encoders enables CM verification,^11^ although *detection templates* are necessary. Typically, we do not always use templates from every gantry position prior to each field irradiation. The acquisition of *templates* involves trade‐offs between time demands, additional imaging dose, performance, and tolerance of possible residual errors at treatment. The responsibility of detecting/assessing residual errors lies with the operators. Using the proposed approach, positional errors were successfully detected and quantified. The $E_{\mathrm{MLDTT}}$ derived by automatic MV tracking was similar to that of manual tracking. This indicates the sensitivity of the approach to detect true residual errors, which is important considering the smaller margins with motion management and higher doses often used in SBRT. It should be stated that the presented approach is not intended as a replacement of x‐ray imaging, but as a complementary solution capable to detect gross errors. MV tracking provides a *direc*t and machine‐independent verification of target‐field alignment, unlike x‐ray and pan/tilt positional feedback (i.e., *indirect*) verification. Using MV tracking would reduce operator workload/reliance and boost confidence in catching overthreshold events (at faster 3 fps) without additional exposure, in contrast to routine (1 fps) x‐ray monitoring. Importantly, it may serve as a failover system when *detection templates* are not available or acquisition failed. Moreover, by exploiting certain tumor histologic/morphologic features, for example, localized and peripherally located (often exhibited by lesions treated with SBRT), one may resort to switching to x‐ray monitoring (as‐needed) only for gantry positions where MV tracking performance is poor or failed.

Multiple aspects of the presented algorithm's methodology can be improved. MV tracking performance is limited by tumor visibility, saliency, and number of traceable features across the tumor region, which in turn depends on motion‐induced artifacts and image quality. It is expected that detector hardware/software advances in the next years will lead to improved image quality. It is noteworthy that the high MV tracking success may be attributed to the cohort selected for MLDTT treatment, which constitutes a potential bias in our analysis. An occlusion coefficient was defined to approximate the expected MLC‐related occlusion. In principle, occlusions can be derived from TPS. However, these may not be representative of the actual due to residual inter‐/intrafraction uncertainties. The tumor visibility‐to‐MLC occlusion ratio was strongly linked to MV tracking success and performance. Similarly, TPS inferences (e.g., GTV contour, MLCs positions) may be utilized, for example, to limit feature matching range within expected confines. Routinely, we limit the number of CPs/MUs in IMRT at plan optimization, minimizing small segments per field. Moreover, 3DCRT is preferred over IMRT due to the increased complexity and missing departmental patient‐specific verification of IMRT plans—the actual motion is available as treatment is being delivered. However, even with cautious planning, it may be challenging to minimize or avoid occluding the target completely. Complexity reduction maneuvers (e.g., aperture optimization) are to play a key role in this regard, preserving an increased amount of tumor (region) visible (unoccluded) within the BEV.^39^ The algorithm, as presented, is strictly limited to determining 2D rigid in‐plane translations of the target only, although the BEV perspective is regarded as sufficient.^25^ Our study re‐emphasizes the continuing utility of MV imaging and serves as further evidence in support of routine intra‐treatment verification and online QA protocols. This will not only complement non‐invasive lung SBRT strategies on dedicated platforms like the Vero4DRT, but generally broaden access to more accurate and precise treatment deliveries on conventional linac‐based setups. Our approach can be readily transferred to other platforms in a similar way as existing template‐based methods for position monitoring,^36^, ^37^, ^40^ supporting ITV‐based, breath‐hold, or gated deliveries. The algorithm's output corresponding to ITV‐based delivery on a TrueBeam accelerator can found in Supporting Information S4. We envision a clinical workflow beginning with the acquisition of gantry angle‐specific portals and training images. This is analogous to stereo‐ and monoscopic approaches, which rely on prior kV imaging for training. DRRs can help select tumors and angles suitable for MV tracking prior to treatment.^27^ Meaningful tolerances including a temporal and positional criterion (e.g., positional error should not exceed 3 mm for more than 500 ms) should be in place. During treatment, the algorithm would operate in the background passively verifying target position using the imager frame feed, without interfering with the clinical workflow. Upon detection of an overthreshold event, a request is invoked for an action (e.g., beam‐on hold, repositioning). This would facilitate some form of image guidance, compared to no *direct* verification at all. This would also complement surface‐guided motion management methods—increasingly being clinically adopted.^8^ A prospective assessment and development of QA procedure, similar to the work by Mueller et al.^40^ would be the next step.

## 5 CONCLUSIONS

A megavoltage image‐based soft‐tissue feature tracking approach for independent and complementary intrafraction motion monitoring in non‐invasive lung SBRT has been proposed and validated based on realistic clinical scenarios. MV tracking was successful for 89.7% of unmodulated (3DCRT) and 82.4% of modulated CPs/SFs (IMRT and field‐in‐field 3DCRT) field deliveries. The mean MV tracking accuracy was 1.2 ± 0.3 mm (pan) and 1.4 ± 0.8 mm (tilt), respectively, and a 2D accuracy of 1.8 mm. The MV‐measured geometric uncertainty of MLDTT treatment was 1.2 ± 0.6 mm (pan) and 1.7 ± 0.9 mm (tilt), compared to 1.1 ± 0.5 mm and 1.5 ± 0.8 mm based on ground truth manual tracking. MLDTT effectively accounted for intrafraction tumor motion. However, caution should be taken with regard to latent changes in the internal/external correlation and breathing dynamics which emphasizes the clinical significance of independent verification, and thus the presented work.

## CONFLICT OF INTEREST STATEMENT

The authors declare no conflicts of interest.

## Supporting information



Material S‐1. Video of output of MV tracking and MLC occlusion handling for during IMRT delivery for one field. The detected tumor position is represented by the blue bounding‐box.

Material S‐2. Video of output of MV tracking under stringent conditions, namely reduced image contrast, considerable peak‐peak motion and anatomy (diaphragm, heart) obstruction.

Material S‐3. Summary of geometric uncertainty of MLDTT by means of automatic and manual MV tracking.

Material S‐4. Video of output of MV tracking approach applied to a conventional accelerator during ITV‐based delivery.
