# Supplementary material for: Megavoltage intrafraction monitoring and position uncertainty in gimbaled markerless dynamic tumor tracking treatment of lung tumors
Source: Med Phys. 2025 Apr 3;52(6):4657–74. doi: 10.1002/mp.17740 (PMC12149710; doi:10.1002/mp.17740)
Supplement: Supplementary file 3 — Material S‐3. Summary of geometric uncertainty of MLDTT by means of automatic and manual MV tracking. [file MP-52-4657-s003.docx]

**Supplementary Table S-3.** Summary of geometric uncertainty in MLDTT treatment in the pan and tilt image plane of the megavoltage imager by means of automatic and manual tracking. For comparison, the geometric uncertainty of the treatment without motion compensation (non-MLDTT), $E_{non-MLDTT}$, was also determined:

$E_{non-MLDTT}= \sqrt{\frac{\sum_{t}^{N} \left( p^{(APER)}\left( 0 \right)-p^{(TAR)}\left( t \right) \right)^{2}}{N}}$

where $p^{(APER)}\left( 0 \right)$ is the aperture center position corresponding to reference frame used for MV tracking algorithm initialization. This may be viewed as the field aperture remaining (static) at the zero position throughout dose delivery.

|  |  |  |  | Mean rmse | |  | M | |  | Σ | |  | σ | |  | 2.5Σ + 0.7σ | |
| --- | --- | --- | --- | --- | --- | --- | --- | --- | --- | --- | --- | --- | --- | --- | --- | --- | --- |
| Motion compensation |  | MV tracking |  | Pan | Tilt |  | Pan | Tilt |  | Pan | Tilt |  | Pan | Tilt |  | Pan | Tilt |
|  |  |  |  |  |  |  |  |  |  |  |  |  |  |  |  |  |  |
| Non-MLDTT |  | Auto |  | 1.8 | 5.1 |  | 1.5 | 3.4 |  | 1.6 | 4.2 |  | 1.4 | 4.2 |  | 2.7 | 16.6 |
| MLDTT |  | Auto |  | 1.2 | 1.8 |  | 0.9 | 1.3 |  | 0.5 | 0.7 |  | 0.8 | 1.2 |  | 1.8 | 2.5 |
|  |  | Manual |  | 1.1 | 1.8 |  | 0.8 | 1.2 |  | 0.4 | 0.6 |  | 0.6 | 0.9 |  | 1.3 | 2.2 |

Group mean error (M), systematic error (Σ), random error (σ), and margins estimated using van Herk’s formalism, given in millimeters (mm).

**Supplementary Video S-4.** Output demonstrating the transferability of the presented MV tracking approach to TrueBeam platform (Varian, Palo Alto, USA) at Freiburg Hospital. The lung cancer patient received SBRT treatment in free breathing with a fractionation scheme of 10 Gy/fraction in 5 fractions, prescribed to the 95% IDL, planned using Eclipse TPS. The tracked tumor position on the consecutive frames is depicted by the blue bounding-box.


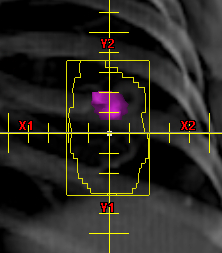
GTV characteristics:

- Volume: 3.2 cm^3^
- Diameter: 1.3 – 1.6 cm
- Amplitude (p-p): 2.5 cm (S-I)

Treatment technique:

- 3D CRT
- 6MV FFF

DRR with overlaid GTV and obstructing ribs
